# The impact of computed radiography and teleradiology on patients’ diagnosis and treatment in Mweso, the Democratic Republic of Congo

**DOI:** 10.1371/journal.pone.0227773

**Published:** 2020-01-15

**Authors:** Iona Crumley, Jarred Halton, Jane Greig, Lucien Kahunga, Jean-Paul Mwanga, Arlene Chua, Cara Kosack

**Affiliations:** 1 Diagnostic Network, MSF International, Amsterdam, Netherlands; 2 Manson Unit, MSF, London, England, United Kingdom; 3 Hôpital General de Reference, Mweso, North Kivu, Democratic Republic of Congo; 4 Diagnostic Network, MSF International, Geneva, Switzerland; Johns Hopkins School of Medicine, UNITED STATES

## Abstract

**Introduction:**

High quality diagnostic imaging can provide increased diagnostic accuracy and help guide medical decision-making and management, however challenges for radiology in resource-limited settings are numerous. Diagnostic imaging and teleradiology have financial and logistical implications, so evidence of impact is crucial. We sought to test the hypothesis that the implementation of computed radiography with teleradiology consultation support will significantly change diagnoses and treatment plans in a resource limited setting.

**Method:**

Paired before-after study to determine the therapeutic impact of an add-on diagnostic test. ‘Preliminary Plan’ and ‘Final Plan’ forms allowed direct comparison of diagnosis and treatment plans at initial consultation and following radiography and teleradiology. Consecutive consenting patients were included until the sample size (600) was reached. Changes in both diagnosis and treatment plan were analysed in the whole cohort, with sub-analyses of children aged <5 years, and cases of chest radiography.

**Results:**

Final analysis included 536 cases. Diagnosis changed following radiography and teleradiology in 62% of cases, and treatment plans changed in 61%. In chest radiography cases, 70% of diagnoses and 62% of treatment plans changed, while in children <5 years 66% of diagnoses and 58% of treatment plans changed.

Reduced final treatment plans were most common for exploratory surgery (72% decrease), surgical orthopaedic intervention (62% decrease), and TB treatment (52% decrease), allowing more conservative medical or surgical management in 61 cases. Increased final treatment plans were highest in the orthopaedic and interventional surgery and referral categories. Of 42 cases requiring interventional surgery in the final plan, 26 (62%) were identified only after radiography and teleradiology. 16 additional cases were indicated for orthopaedic surgery, 10 cases required patient transfer, and TB treatment was indicated in 45 cases. A change in the original prescription plan occurred in 41% of 536 cases, with one or more prescriptions stopped in 28% of all cases.

**Conclusion:**

We found that computed radiography with teleradiology had significant clinical value in this resource-limited setting, with the potential to affect both patient outcomes and treatment costs through providing improved diagnostics and avoiding unnecessary treatments and medications.

## Introduction

It is widely acknowledged that high quality diagnostic imaging can provide increased diagnostic accuracy and help guide medical decision-making and management, with positive correlation in outcomes for patients, providers and wider health-care systems [1]. However, there exists a ‘radiology divide’ between low- and middle-income countries, and high-income countries, particularly in remote areas due to the lack of access to safe and reliable diagnostic imaging [2,3,4].

The challenges for radiology in resource-limited settings are numerous. Where diagnostic imaging exists, insufficient supply and maintenance, and inappropriate equipment donation often lead to outdated and non-functional units [1]. Many radiology departments in resource-limited settings still use film and chemical development, where consumable rupture can be a frequent challenge to continuous service provision. Associated problems include lower image quality, higher radiation dose to patients and improper waste disposal of chemical and film consumables. [1]

Several developments in technology have improved the quality of diagnostic radiology worldwide, in both production and interpretation of images. Of particular note are advances in digital radiography, now near ubiquitous in western settings providing increased image quality and reduced radiation dose, and in teleradiology, allowing expertise to reach remote or underserved settings, improving medical education, reducing referrals and costs to health care systems, and positively impacting patient outcomes [5].

In many settings, formal radiology education is omitted from basic medical training; radiologists are near non-existent and radiography not a recognised profession [1,6]. Therefore, untrained staff performing radiographic procedures may result in images of low diagnostic quality, with the limited capacity of medical staff to interpret radiographs correctly further reducing the diagnostic merit of the examinations.

Since 2010, Médecins sans Frontières has operated a teleradiology service as part of its wider store-and-forward telemedicine platform, allowing field sites access to consultant radiologist reporting [7,8]. Radiology is a medical specialty that adapts particularly well to remote specialist diagnosis [9], with digital radiographs having the added advantage of being easily electronically transmitted.

Few studies have explored the feasibility of computed radiography and telemedicine in a resource-limited setting [10], and aimed to show a measurable potential effect on diagnosis and treatment through implementation of these tools [11]. Providing measures of the impact of computed radiography and telemedicine in such contexts could be important for decision makers, particularly in countries where needs are high, yet infrastructure is low and there is constant competition for limited resources and funding.

This study therefore sought to test the hypothesis that the implementation of computed radiography with teleradiology consultation support will significantly change diagnoses and treatment plans in a resource-limited setting.

## Methods and materials

### Study setting

Médecins Sans Frontières has been present in the eastern provinces of North and South Kivu since the early 1990’s. A lack of health care infrastructure and difficult terrain, together with ongoing conflict, has limited access to basic services for large sections of the population. [12,13,14,15].

Hôpital General de Reference (HGR) situated in Mweso, North Kivu, is a Ministry of Health (MOH) run, Médecins Sans Frontières supported, facility providing access to free primary and secondary level health care, to a target population of approximately 145 000. In 2013 over 138 000 out-patient department (OPD) consultations were carried out, and nearly 6000 patients were admitted to the hospital, whose services include surgery, internal medicine, paediatric care, reproductive health, nutrition and psychiatry. [16].

Prior to 2015, radiography facilities were not available within several hours drive. Diagnostic services were limited, and diagnosis relied on patient history, clinical examination, and basic laboratory tests. In 2015 over a period of three months, a computed radiography (CR) system was installed in HGR and extensive training provided on its use to four existing medical local staff members who would operate the equipment. Training on image interpretation and justified referring for physicians was provided. In addition, the telemedicine platform was introduced on site.

### Study procedure

The study was conducted between September 2015 and December 2017, although data collection paused between January 2016 and September 2017. All adult and paediatric patients presenting at HGR, for whom the treating physician considered that radiography and teleradiology consultation may offer additional clinical value, were included in the study. Potential participants were excluded from the study if they refused or subsequently withdrew consent for participation. Consecutive consenting patients were included during data collection periods until the sample of 600 was reached. Written informed consent was obtained from all patients or legal guardians, with study information available in French and Kiswahili. The study (ID 1447) was approved by the Ethics Review Board of Médecins Sans Frontières (Geneva, Switzerland) and the local health authorities in the Democratic Republic of Congo.

On referring a patient for radiography, the treating physician completed a radiology referral form, as well as a ‘Preliminary Plan’ form containing the preliminary diagnosis, treatment plans and prescriptions, based on all information available to them at that point, including any available diagnostic tests except for radiography. The technician completed the requested radiographic examination and posted the clinical details and images to the telemedicine platform, then notified the physician when the radiology report was received. The same physician then reviewed the radiology report, and completed the ‘Final Plan’ form, noting the final diagnosis, treatment plan and prescriptions. These final details were recorded additionally in the patient’s notes.

### Statistical design

The study was designed as a paired before-after study, to determine the therapeutic impact of an add-on diagnostic test [17,18]. The sample size of 500 was based on an estimated change of 15%, with 80% power and a significance level of 5%. Based on the same estimated change and level of confidence, the sample size for patients aged under-five years was 180. To account for this and assuming some loss of usable data due to contextual factors, we aimed to enrol 600 patients in the study.

Data from the ‘Preliminary Plan’ and ‘Final Plan’ forms was double-entered into a database created in EpiData 3.1 software (EpiData, Odense, Denmark), by the study co-ordinator on site and verified by members of the study team based in Amsterdam. Data was exported to Microsoft Excel and analysed using Microsoft Excel and STATA 15.1 (StataCorp, Texas, USA). Baseline characteristics and change in treatment and management were described as counts and percentages with binomial 95% confidence intervals (95%CI) in each category and compared with chi-squared tests.

## Results

The study was conducted over a period of 28 months with repeated interruptions due to security and logistical issues. Of the 600 original paired data records, 64 were excluded because of incomplete data or unresolved errors in the completion of the data forms, leaving 536 cases for analysis. Males constituted 59% of participants, and 25% were under the age of five. Thorax (chest) was the most requested examination (58%), with respiratory illness the most frequently noted clinical indication (50%), followed by questions regarding management for orthopaedics (30%) (Table 1).

**Table 1:** Case characteristic of included data sets.

Description	N = 536
**Age**	
0–4	133
5+	403
**Sex**	
M	315
F	221
**Pulmonary TB**	
Suspected	231
Confirmed	5
Negative	5
Unknown	295
**Extra pulmonary TB**	
Suspected	144
Confirmed*	4
Negative	1
Unknown	387
**HIV-status**	
Positive	66
Negative	34
Unknown	436
**Type of Radiograph**	
Thorax	312
Abdomen	36
Pelvis	26
Extremity upper (right, left)	40
Extremity lower (right, left)	101
Spine (cervical, thoracic, lumbar)	17
Others^¥^	4
**Clinical indication**	
Respiratory illness	267
DD respiratory illness vs. cardiac cause	25
DD for surgical vs. conservative management–orthopedically (e.g. fractures)	158
DD for surgical vs. conservative management–thorax / abdomen	46
Follow-up radiograph for treatment response	18
Others^Ω^	22

DD = differential diagnosis

TB = tuberculosis

*Confirmed by smear microscopy or GeneXpert, or culture result from other external facility.

^¥^ Mandible, Skull

^Ω^ Suspected mass, metastases, suspected extrapulmonary TB (EPTB), osteomyelitis

### Impact on diagnosis and treatment plans

Among all cases, changes were seen between the ‘Preliminary Diagnosis’ and ‘Final Diagnosis’ in 62% (95%CI: 58–66%).

**Fig 1:**
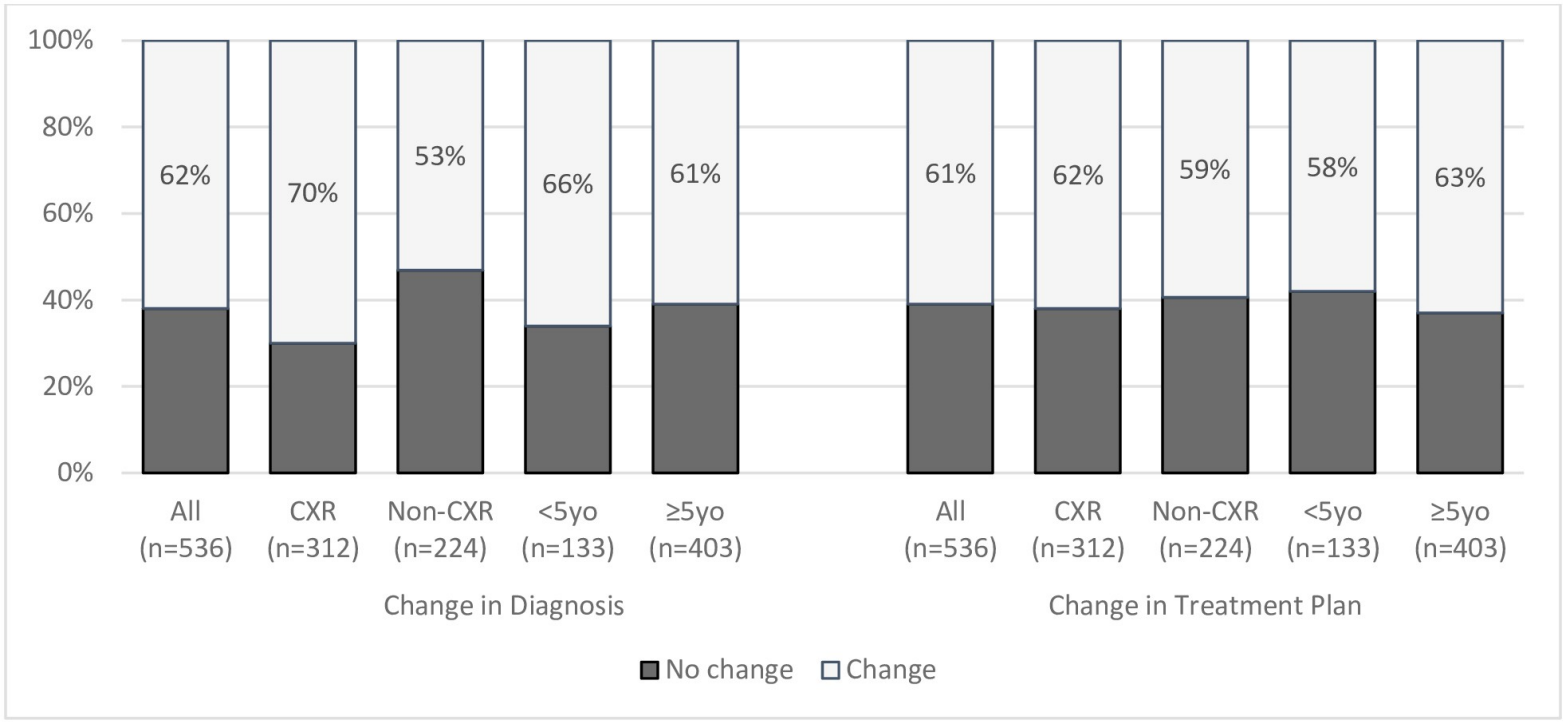
Proportion of cases with change in diagnosis and change in treatment plan as recorded in ‘Preliminary Plan’ and ‘Final Plan’, overall and by sub category. <5yo = Patients under 5 years of age ≥5 = Patients over 5 years of age CXR = Chest Radiograph.

Change in diagnosis was significantly higher for chest radiographs than for other types of radiographs [70% (95%CI: 65–76%) vs. 53% (95%CI: 46–60%); p<0.001], while it was not different in patients <5 years compared to ≥5 years of age [66% (95%CI: 57–74%) vs. 61% (95%CI: 56–66%); p = 0.27] (Fig 1).

The ‘Final Treatment Plan’ was changed in 61% (95%CI: 57–66%) of all cases (Fig 1). The proportion changed was similar for chest radiograph cases and other (non-chest) radiographs [62% (95%CI: 56–67%) vs. 59% (95%CI: 53–66%); p = 0.61], and in patients aged <5 years compared to those aged ≥5 years [58% (95%CI: 49–66%) vs. 63% (95%CI: 58–67%); p = 0.34] (Fig 1).

While the proportion of change in diagnosis is similar to that of change in treatment in the whole cohort (62%, 61% respectively) and the 75% of patients aged ≥5 years (61%, 63%), there was 8% lower proportion of change in treatment than diagnosis in those age <5 years (66% diagnosis, 58% treatment) or having chest radiography (70% diagnosis, 62% treatment).

Amongst cases where a diagnosis was changed, (n = 333) a corresponding change of treatment occurred in 76%. In the cases with no change of diagnosis (n = 203), a change in treatment plan nonetheless occurred in 37% (Table 2).

**Table 2:** Corresponding changes in diagnosis and treatment across entire cohort.

All cases (n = 536)	n	%
Change in diagnosis, change in treatment	254	47
Change in diagnosis, no change in treatment	79	15
No change in diagnosis, change in treatment	75	14
No change in diagnosis, no change in treatment	128	24

### Changes in individual cases in treatment plans

**Fig 2:**
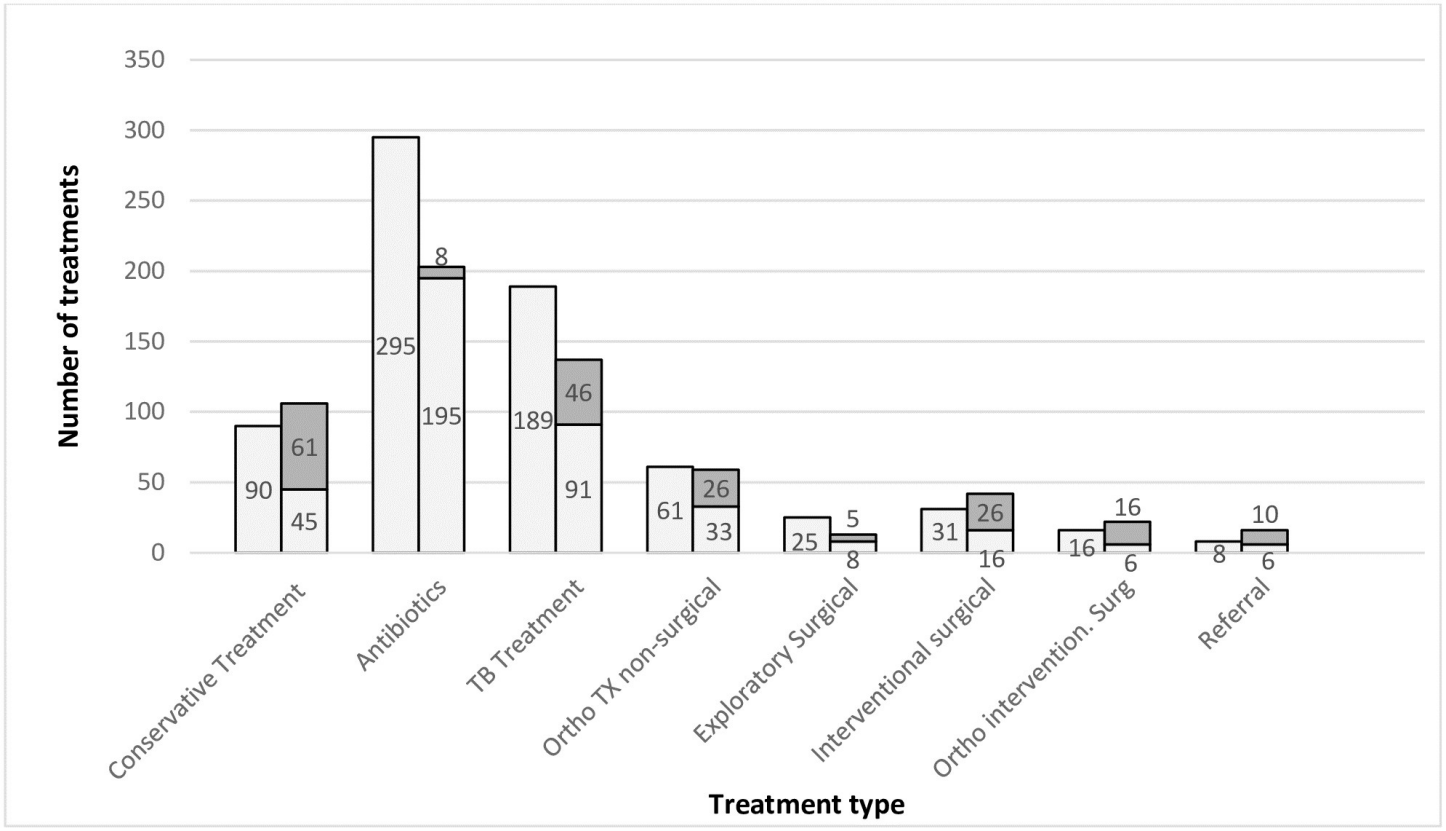
Effect of radiography and telemedicine on individual treatments, showing treatments removed from ‘Preliminary Treatment Plans’ and treatments added in the ‘Final Treatment Plans’. **Column 1**: ‘Preliminary Treatment Plans’: Absolute number of treatments in ‘Preliminary Treatment Plans’. **Column 2**: ‘Final Treatment Plans’: Absolute numbers of treatment figures by category. **Light Grey**: Number of final treatments which were present in the ‘Preliminary Treatment Plan’. (Reduction from column 1 demonstrating decrease in original treatments post radiography and telemedicine.) **Dark Grey**: Absolute number of final treatments which were not present in the ‘Preliminary Treatment Plan’. (That is, treatment added post radiography and telemedicine). **TX** = Treatment **Ortho** = Orthopaedic **Surg** = Surgical.

Changes between the ‘Preliminary’ and ‘Final Plans’ for individual patients were analysed by treatment category (Fig 2). The most substantial decrease in absolute numbers of treatments were in antibiotics (n = 100) and TB treatment (n = 98), with the largest proportional decreases in exploratory surgery (72%), surgical orthopaedic intervention (62%), and TB treatment (52%). In the surgical categories combined (i.e. surgical intervention, orthopaedic surgical intervention and exploratory surgery) 19% (n = 14) changed to conservative treatment.

In the ‘Final Treatment Plans’ 42 cases were scheduled to undergo interventional surgery, of which only 38% were planned for interventional surgery in the ‘Preliminary Treatment Plan’, meaning a further 26 cases were identified in which interventional surgery was indicated as a result of radiography and teleradiology. Similarly, 16 additional cases were indicated for orthopaedic surgery, 10 cases identified requiring patient transfer, and TB treatment was indicated in 45 cases. Conversely, 61 cases which had medical or surgical management in the ‘Preliminary Treatment Plan’ were changed to conservative management after radiography and teleradiology.

**Fig 3:**
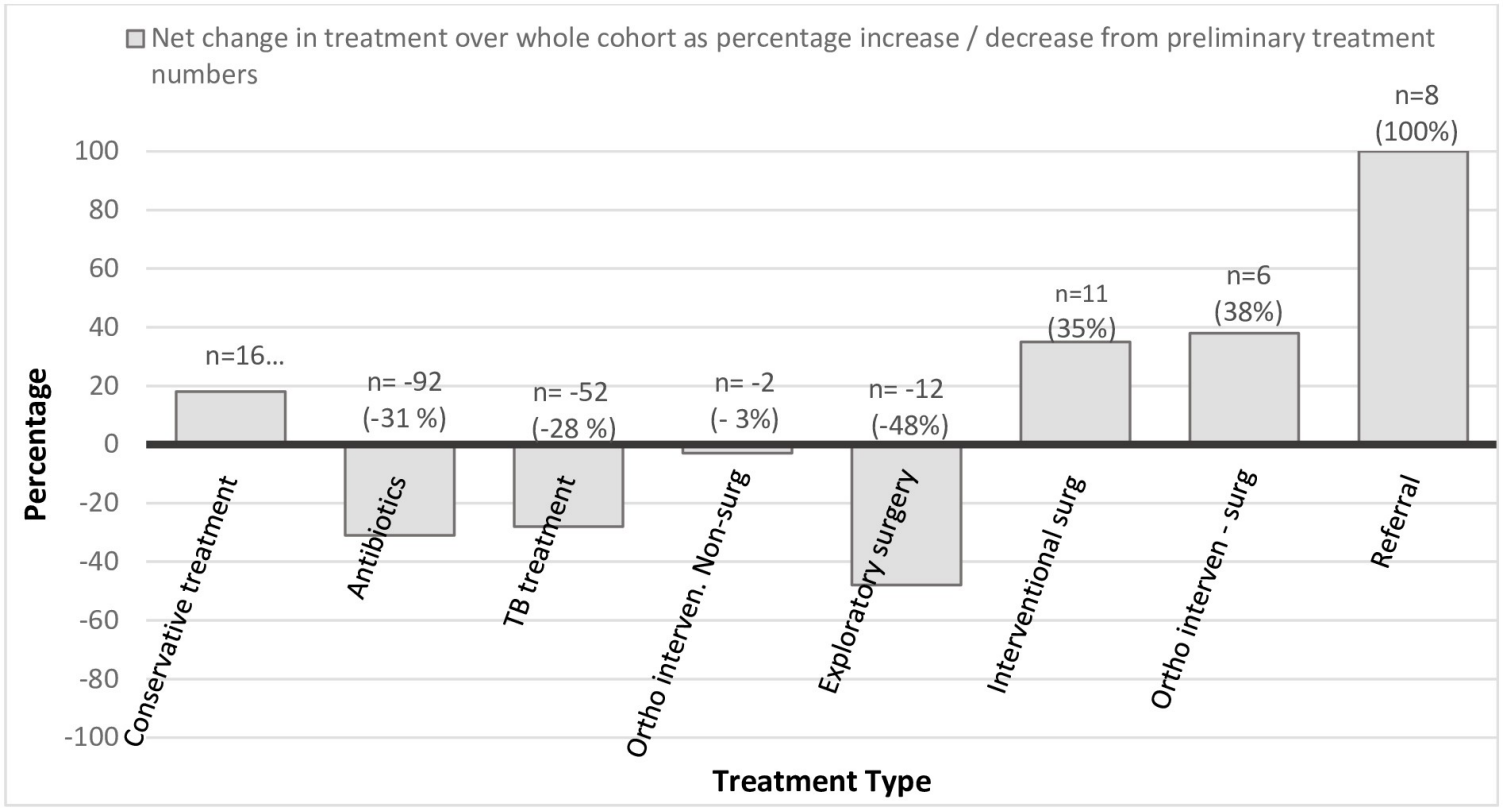
Net change of various treatments across the entire cohort, post radiography and telemedicine, in numbers and as percentage of ‘Preliminary Treatment Plan’.

We also evaluated the net overall change in individual treatments across the entire cohort as absolute number of treatments, and as percentage increase or decrease from the ‘Preliminary Treatment Plan’ (Fig 3). The absolute decrease was largest for TB treatment and antibiotics. The proportion was greatest for exploratory surgery (48%) but was only 12 cases. Net increases were seen in interventional and orthopaedics surgery and referral, as well as conservative management.

### Impact on prescriptions

A change in the original prescription plan was seen in 41% (95%CI: 36–45%) of all 536 cases, with one or more prescriptions stopped in 28%. In patients aged <5 years, 48% (95%CI: 39–57%) of prescription plans were changed, with 36% stopping the prescribed drugs. In chest radiography cases 55% (95%CI: 49–60%) of prescriptions changed, with 39% of cases having prescribed drugs stopped, and 15% additional drugs prescribed (Table 3).

**Table 3:** Changes in prescriptions between ‘Preliminary Treatment Plan’ and ‘Final Treatment Plan’.

	ALL (n = 536)	<5yo (n = 133)	≥5yo (n = 403)	CXR (n = 312)
	%	%	%	%
No change to prescription plan	59	52	62	45
*Some change to prescription plan*	*41*	*48*	*38*	*55*
1 or more drugs from initial prescription stopped	28	36	26	39
1 or more drugs from initial prescription changed	4	2	5	5
1 or more drugs from initial prescription changed in dose or duration	2	2	2	3
1 or more drugs added to initial prescription	10	12	9	15

<5yo = Patients under 5 years of age

≥5yo = Patients aged 5 years or older

CXR = Chest radiography

In terms of individual drug prescriptions (n = 793), 31% were stopped after radiography and teleradiology, of which 65% were antibiotics and 34% were TB drugs. In prescriptions for patients aged <5 years, 30% were stopped (64% antibiotics and 34% TB drugs), and for chest radiography cases, 33% were stopped (68% antibiotics and 29% TB drugs).

Assessment by drug category revealed that only 50% of all TB treatment prescriptions were continued unaltered post radiography and teleradiology, with 44% stopped, and 6% altered in regimen.

## Discussion

Our study results demonstrated that, as hypothesised, access to computed radiography and teleradiology in a low resource setting resulted in significant changes to diagnosis (62%) and management plans (61%), suggesting important benefits in patient care.

We found a significantly higher proportion of change in diagnosis for chest radiography compared to non-chest radiography, but this did not equate to a difference in change of treatment plans in these 2 groups. There was no difference in proportion of change for either diagnosis or treatment by age group (<5 vs. ≥ 5 years).

The greater change in diagnosis with chest radiography perhaps relates to a widely accepted use of chest radiography in the detection and diagnosis of lung disease, and its promoted use as a tool which can aid in the detection of TB [19,20]. Further, this might relate to the difficulties facing clinicians in making a confident diagnosis where patients present with chest symptoms, an effect likely amplified in a resource-limited setting where TB is prevalent, there is limited access to other confirmatory diagnostic tests, and treatments are often commenced empirically. While smear microscopy was available for TB testing, GeneXpert molecular testing for TB and Rifampicin resistance only became available during the latter half of the data collection, and while culture was available it was rarely used due to difficulties in collecting and transporting samples.

The lack of a corresponding higher rate of treatment change in the chest radiography group may reflect that many antibiotics prescribed for suspected pneumonia were not stopped following a negative radiograph, due to various protocols regarding antibiotic use including protocols on the absence of a definitive diagnosis for the causative organism for pneumonia.

Gross musculoskeletal injuries may be clinically obvious without radiography, therefore change in diagnosis may be unlikely, but the value of radiography guiding appropriate management is reflected in the greater impact on treatment.

The overall net result post radiography and teleradiology in both prescription and treatments was that more prescriptions were stopped than added, and more treatments avoided than supplemented. In a resource-limited setting, where, with limited other diagnostic tests a tendency towards empirical treatment may dominate, this effect is important. In the case of antibiotics and anti-TB treatment, the issues of overprescribing and the impact on resistance must also be considered. [21] Considering the number of initial individual prescriptions (n = 793), the overall reduction seen equates to many avoided medications.

Our sub-analysis on treatment type also revealed a large proportional impact on potential surgical cases. For those cases of unnecessary surgeries averted, this potentially translates to avoidance of high costs and patient risks especially those associated with surgical intervention and transfer in a low-resourced or conflict setting. For surgeries added following radiography and teleradiology, while we have no data on the eventual patient outcome, the positive impact of early recognition of the need for surgical intervention is well recognised. [22] Equally, for hospital transfers, faster identification of cases requiring a higher level of care, and diagnostic evidence to justify the transfer, may have enhanced patient outcome.

The installation and maintenance of radiology equipment is resource intensive. [1] Particularly in settings with many competing priorities, it is important to demonstrate the potential return on such an investment, not only in terms of improved diagnosis but also in improved patient management, and a corresponding potential for cost saving through impact on prescriptions, surgical interventions, bed occupancy and referral rates. In addition, it should be considered that those cases where no change occurred in either diagnosis or treatment (24%) may still have benefitted from the confirmation of suspected diagnosis. In resource-limited settings where patient access to treatment and follow-up care may be extremely difficult, and in some settings have a direct as well as indirect cost to patients, the value of this should not be underestimated.

We did not systematically assess user acceptance of the new services, but anecdotal feedback from users during the study was very positive on the impact of radiography and teleradiology to assist them in the diagnosis and treatment of patients, with many users commenting that they would have sent the radiograph to teleradiology, even had they not been required to do so as part of the study protocol. Indeed, reviewing programmatic case numbers during the 4 months following completion of the study indicated that the staff at HGR continued to send around 55% of all radiography cases to telemedicine.

### Limitations

Several factors should be considered which might have influenced the reported data. While full training on the study protocol and completing the data collection forms was implemented on site, the hypothetical element to the ‘Preliminary Plans’ which imagined a scenario in which radiography and telemedicine was not available may have been difficult for staff to strictly adhere to. It may be that ‘Preliminary Plan’ forms were completed to include all potential treatments for the differential diagnosis, rather than only the treatments which would have been initiated at that point were radiography not available. Future planned treatments (e.g. TB treatment if no response to initial antibiotics) may have been removed in the ‘Final Plan’ as a result of radiography, but as they had not yet been commenced and may never have been with adequate clinical response, it could be argued that our method overestimated the number of treatments or prescriptions stopped.

The availability of other diagnostic tools and tests may have influenced changes in diagnosis and management. At the time of the study, HGR also had diagnostic laboratory capabilities including basic virology for HIV rapid diagnostic test, CD4 count and hepatitis B rapid diagnostic test, basic haematology and parasitology. During the study, an immediate treatment plan in the best interest of the patient was put in place using all available tools and tests, in addition to computed radiography. Inevitably, if the results of these laboratory tests were received between the ‘Preliminary Plan’ and ‘Final Plan’, they may have influenced the final diagnosis and treatment noted. We are unable to estimate the scale of this effect as we did not predict the availability of new diagnostics so had not sought permission to collect and analyse results of other diagnostic tools, nor did we ask on the ‘Final Plan’ form why any change was made.

Further, treatment decisions were made on the treating physician’s interpretation of the radiograph without waiting for teleradiology results in all cases where this was in the best interest of the patient. It may have been almost impossible in some such cases, to define one clear preliminary and one final diagnosis and plan, whilst disregarding the impact of the physician’s own interpretation of the radiographs. No data was recorded on the physician’s interpretation of the radiograph, which would have allowed us to separate the very distinct tools of computed radiography and telemedicine radiology reporting, or to assess the benefit of each individually. Such data would have added further information on the individual values of radiography and teleradiology, which may be very different in the different clinical scenarios. In addition, as the study was not designed to include follow up data, we cannot relate the changes to impact on outcomes.

Although data collection was intended to be continuous, this was impossible in practice for reasons related to the context. The data was collected over a period of over 2 years, during which there was some staff turnover, therefore data collection may have suffered from lack of continuity.

Further, although we attained the intended total sample size, the intended sample size of patients aged <5 years was based on an expected proportion of all patients, which was not achieved within the final sample.

## Conclusion

Access to computed radiography and teleradiology had significant impact on diagnosis and treatment in this study cohort in a general referral hospital in the Democratic Republic of Congo. We conclude that computed radiography had significant clinical value in this resource-limited setting, with the potential to affect patient outcomes through better diagnosis and associated treatment plans, including avoiding unnecessary treatments and medications.
