# Correction: The impact of computed radiography and teleradiology on patients’ diagnosis and treatment in Mweso, the Democratic Republic of Congo

**DOI:** 10.1371/journal.pone.0299648

**Published:** 2024-02-23

**Authors:** Iona Crumley, Jarred Halton, Jane Greig, Lucien Kahunga, Jean-Paul Mwanga, Arlene Chua, Cara Kosack

The Funding statement and Competing Interests statement are incorrect. The correct statements are:

**Funding:** Funding for this study was granted internally by the Innovation Fund of Médecins Sans Frontières (MSF). MSF research is undertaken as part of operations and financial support for this research was absorbed by MSF budgets. MSF provided support for this study in the form of salaries for all authors. The specific roles of the authors are articulated in the ‘author contributions’ section. MSF was involved in the study design, data collection and analysis, decision to publish, and preparation of the manuscript.

**Competing Interests:** The authors have read the journal’s policy and have the following competing interests to declare: MSF provided support for this study in the form of salaries for all authors. This does not alter our adherence to *PLOS ONE* policies on sharing data and materials. There are no patents, products in development or marketed products associated with this research to declare.
